# History of Reading Struggles Linked to Enhanced Learning in Low Spatial Frequency Scenes

**DOI:** 10.1371/journal.pone.0035724

**Published:** 2012-04-27

**Authors:** Matthew H. Schneps, James R. Brockmole, Gerhard Sonnert, Marc Pomplun

**Affiliations:** 1 Science Education Department, Harvard-Smithsonian Center for Astrophysics, Cambridge, Massachusetts, United States of America; 2 Department of Psychology, University of Notre Dame, Notre Dame, Indiana, United States of America; 3 Department of Computer Science, University of Massachusetts at Boston, Boston, Massachusetts, United States of America; Nothwestern University, United States of America

## Abstract

People with dyslexia, who face lifelong struggles with reading, exhibit numerous associated low-level sensory deficits including deficits in focal attention. Countering this, studies have shown that struggling readers outperform typical readers in some visual tasks that integrate distributed information across an expanse. Though such abilities would be expected to facilitate scene memory, prior investigations using the contextual cueing paradigm failed to find corresponding advantages in dyslexia. We suggest that these studies were confounded by task-dependent effects exaggerating known focal attention deficits in dyslexia, and that, if natural scenes were used as the context, advantages would emerge. Here, we investigate this hypothesis by comparing college students with histories of severe lifelong reading difficulties (SR) and typical readers (TR) in contexts that vary attention load. We find no differences in contextual-cueing when spatial contexts are letter-like objects, or when contexts are natural scenes. However, the SR group significantly outperforms the TR group when contexts are low-pass filtered natural scenes [F(3, 39) = 3.15, p<.05]. These findings suggest that perception or memory for low spatial frequency components in scenes is enhanced in dyslexia. These findings are important because they suggest strengths for spatial learning in a population otherwise impaired, carrying implications for the education and support of students who face challenges in school.

## Introduction

It has long been recognized that memories for the structure and layout of a scene, whether real or imagined, can constitute a framework for housing decontextualized memories for discrete objects, numbers, or words that can powerfully augment abilities for episodic memory [1]. And thus, while scene memory is a vital life function in many applications for all individuals, it is perhaps especially important among those whose abilities for episodic memory are limited and who may depend on strengths for scene memory to compensate for impairments in other areas. Dyslexia is a neurological learning disability characterized by lifelong struggles with reading and spelling that are unexpected given a person's capabilities in other cognitive domains [2]. People with dyslexia exhibit phonological processing deficits, together with impairments in working memory and short term memory [3], that impair the episodic recall of words, dates, and numbers. Therefore, people with dyslexia stand to benefit from strategies that use spatial encoding to augment memory, and they may make use of such strategies to achieve at high levels despite struggles in various cognitive domains. Supporting this hypothesis, cases of nonverbal giftedness in dyslexia are documented [4], and those with dyslexia include numerous examples of highly successful individuals including the Nobel laureates Carol W. Greider and Baruj Benacerraf [5], [6]. If such individuals use spatial learning strategies to compensate for difficulties encoding memories phonologically, we would expect to see evidence of exceptional facility for spatial learning in dyslexia.

Contextual cueing [7] is a research paradigm often used to provide a measure of spatial learning. In this task, participants search for a target hidden in a visual display, and the speed of search in spatial contexts that are novel is compared with the speed of search in contexts that have been previously searched. Response time is typically speeded up as repeated displays are learned, and this search benefit can be ascribed to spatial learning. Such learning has been demonstrated across a wide range of contexts, ranging from arrays of letters [7] to real-world scenes [8], and is driven by a variety of factors invoking processing in the central and peripheral visual fields [9]–[12]. However, when contextual cueing was used to investigate abilities for spatial learning in people with dyslexia [13]–[15], the expected advantages for spatial learning were not reliably observed.

We suggest that previous studies of contextual cueing in dyslexia failed to reveal advantages for spatial learning because these experiments were confounded by task-related demands for focal attention. People with dyslexia exhibit deficits in focal attention [16]–[18], which can even be observed in preschool children at risk of dyslexia prior to the acquisition of reading [19]. Hence, previous instantiations of contextual cueing paradigms have likely been ill suited tests of spatial abilities among individuals with dyslexia. Specifically, in the previous studies, letter-like objects were used for the spatial context. Subjects searched for a target while simultaneously performing a discrimination task to distinguish target objects (T shape) from similarly shaped background objects (L shapes) that were subtly doglegged to increase difficulty and slow the search. We suggest that the increased cognitive load at the fovea induced by this discrimination task evokes an inhibitory neurological response that diminishes sensitivity in the periphery [20]. Studies of contextual cueing during covert search show that the periphery plays an important role in spatial learning for contexts composed of letter-like forms [12]. Therefore, if peripheral sensitivity is inhibited in dyslexia by heightened cognitive demands during search, this effect could mask potential advantages for spatial learning and thereby account for the lack of reliable findings [14].

Here, we investigate the possibility that spatial learning advantages in dyslexia will be observed if the task is matched to strengths observed in this group. A number of authors stress a distinction between systems for focal attention and those for distributed spatial attention [21], [22]. Distributed attention is thought to contribute to scene perception by enabling the extraction of a gist, a rapidly obtained initial hypothesis about the scene's identity and global layout [23] that is then refined through shifts of focal attention [24]. While focal attention is impaired in dyslexia [25], [26], other evidence suggests that distributed attention is unimpaired and is perhaps even enhanced. For example, the recognition of impossible figures, which is thought to depend on the holistic integration of long-range spatial information, is faster among people with dyslexia, compared with typical readers [27]. Visuospatial advantages in dyslexia have also been suggested in letter identification tasks where letters are flashed simultaneously at fixation and in the periphery, a task that requires rapid deployment of spatially distributed attention. Recognition accuracy of letter pairs is enhanced in dyslexia when peripheral letters are presented at eccentricities >7.5° [28]–[34]. Similarly, those with dyslexia are reported to respond more rapidly to an unattended peripheral flash when the flash occurs at eccentricities >8° [35], [36]. Collectively, these studies link dyslexia to advantages for distributed forms of spatial attention, typically in circumstances where peripheral information is important.

It has been suggested that these seemingly contradictory observations of co-occurring deficits and advantages in visual processing linked to dyslexia can be understood in a framework that considers the central and peripheral visual fields (here defined as divided at roughly 8° eccentricity) to be structurally segregated and differentiated by their anatomical and functional characteristics [37]. For example, while the center is helpful in searching for small objects [38], the periphery is better optimized for rapid discriminations [39]. Such functional differences can be traced to eccentricity dependent differences in cortical anatomy that originate at the retina [40], [41] and that in turn project to the visual cortex so as to largely preserve the retinotopic organization. As a consequence, the functionalities of the center and the periphery remain grossly segregated throughout the brain [42]. If we take as an axiom that the center and periphery can be considered separate yet complementary visual systems, the degree to which individuals vary in their abilities to make use of each source of information can be characterized by using a semi-quantitative descriptor called the periphery-to-center ratio (PCR) [37]. In this perspective, PCR is high in many with dyslexia, which means that information in the peripheral visual field is favored over information in the center. This is consistent with accounts for focal attention deficits that impair search, but also with advantages for distributed attention that enhance spatial learning.

**Figure 1:**
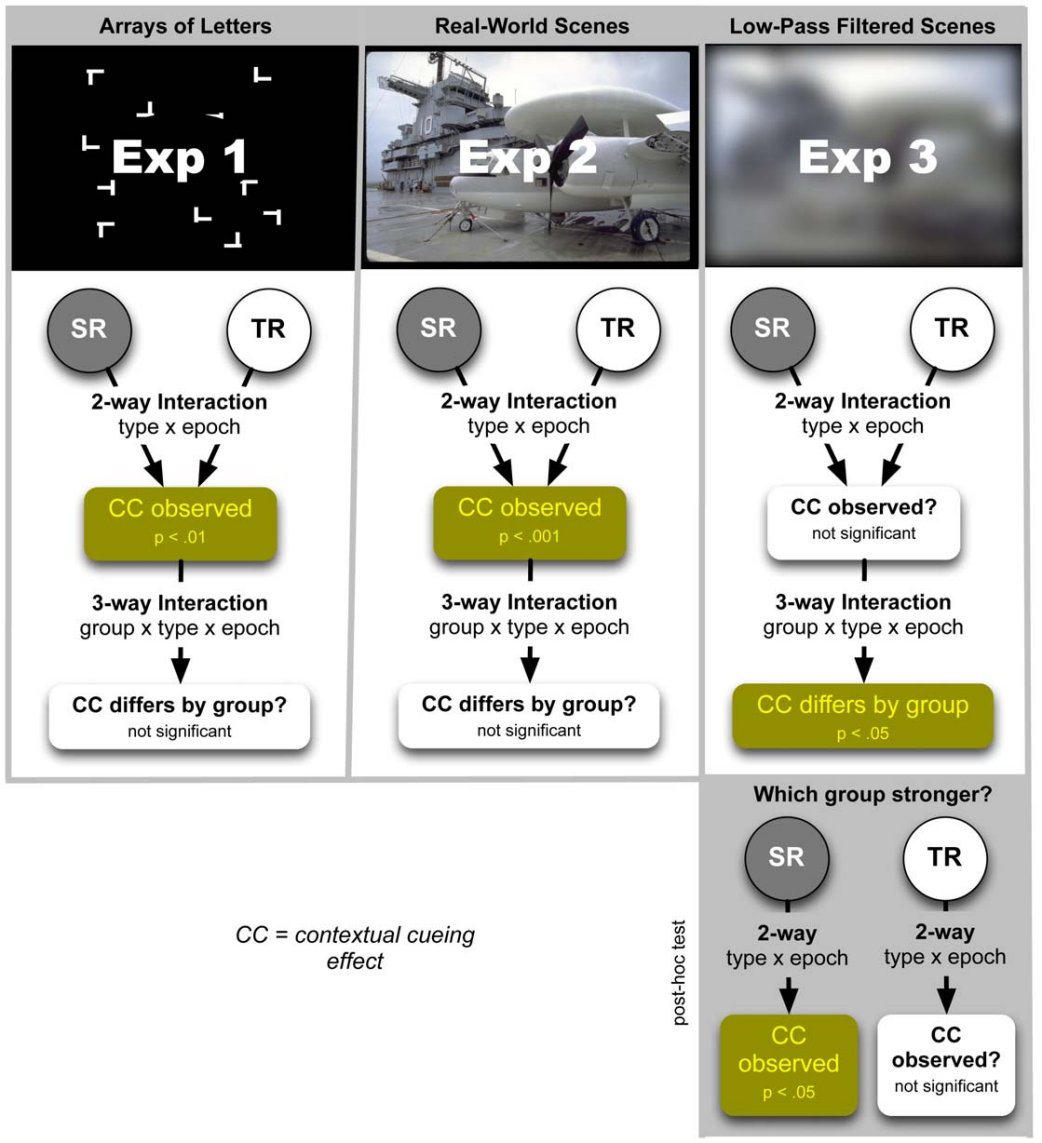
Schematic of experiments, analysis, and results. Contextual cueing is observed in both TR and SR groups when letter-like objects (Experiment 1) or natural scenes (Experiment 2) are used as spatial contexts. Here, the response in the two groups is indistinguishable. However, when low-pass filtered scenes are used as the spatial context (Experiment 3) a significant group interaction is observed. Further analysis reveals significant scene learning in the SR group not evident in the TR group.

Keeping in mind deficits in focal attention and enhanced reliance on peripheral vision among individuals with dyslexia, we propose that advantages for spatial learning in dyslexia would be more likely evident if (a) a simple feature search replaced the previously used complex discrimination task to reduce cognitive load at the fovea, and (b) the contextual background to be learned made maximal use of long-range information sensitive to the periphery. While the periphery contributes to spatial learning when contexts are letter-like forms [12], in such contexts spatial learning is restricted to content local to the target [9]. In contrast, when contextual cueing is performed in natural scenes spatial learning is observed to be strongly influenced by long-range global information integrated across the scene [10]. Therefore, if contexts consisting of letter-like forms are replaced by natural scenes, and a simple feature search (to locate an L or T in the scene background) replaces the more complex discrimination task often used, spatial learning advantages in dyslexia may be more evident than they have been in previous studies [13]–[15]. These cases are explored in Experiments 1 and 2. Lastly, we suggest that spatial learning advantages in dyslexia are more likely to be detected if contexts are low-pass filtered scenes. While the center is exquisitely sensitive to high-spatial frequencies, the periphery is relatively blind to this information. Therefore, if natural scenes are low-pass filtered, the loss of high spatial frequency information would extract a greater toll on functionalities of the center. In the PCR framework, this would bias spatial learning in favor of those with dyslexia, a hypothesis explored in Experiment 3. (The experiments undertaken are schematically summarized in Figure 1.)

## Experiment 1: Arrays of Letters

### Methods

#### Ethics Statement

The Institutional Review Boards of Harvard University and Landmark College approved this study. All participants signed an informed consent form and were paid $20 for their participation in this experiment.

#### Participants

In this experiment a group of 10 struggling readers were randomly selected from a pool of 19 students recruited for these studies from Landmark College in Putney, VT. Landmark is a two-year liberal arts institution exclusively for students with learning impairments such as dyslexia. Students attend this school only if past instructional histories suggest severe learning impediments that were not remediated in previous grades and that would place these students at risk if enrolled directly in a traditional college environment without further support. All participants in our pool had childhood histories of serious reading impairments that persisted into adulthood, and had been assigned a diagnosis of dyslexia by the Landmark psychologist. Evidence of reading struggles consistent with dyslexia were ascertained by examining records of psychological testing on file at the school to verify that reading subtests from well-established achievement tests (WJ-III Achievement [43] or WIAT-II [44]) were significantly below ability tests (WAIS-III [45] or WJ-III Cognitive [46]), as is characteristic of dyslexia. Landmark students meeting these criteria were included in the study if no additional history of neurological disorders was evident. Table 1 summarizes behavioral data for each student in the pool who participated in these experiments, obtained from records on file at the school. Privacy conditions for access precluded our carrying out tests to obtain additional behavioral measures in this group. Hence, we have no information about effective subtypes of dyslexia represented in the sample and therefore refer to this group simply as “struggling readers” (SR). A control group of typical readers (TR) included 19 college students from Harvard University who had no history of dyslexia, attention deficit disorder (ADHD), or other neurological disorders. Harvard is ranked among the premier institutions of higher learning in the United States, and admission is highly competitive. Therefore, there is a strong selection bias in both the SR and TR groups, in that these are likely to represent the extremes in reading ability among college students. All Harvard recruits were administered questionnaires to check for possible histories of learning difficulties. The Adult Reading History Questionnaire (ARHQ), developed by Lefly and Pennington [47], based on [48], includes questions about learning letter names, learning to spell, reading speed, effort needed to succeed, and verbal short-term memory performance, etc. Lefly and Pennington (2000) reported the internal consistency (alpha) of the original ARHQ to be .94, and the test–retest reliability over a 2-year period to be .87. Volunteers were admitted to the study pool only if they scored above a cutoff of 0.30 on the ARHQ. In addition, the 6-question World Health Organization Adult ADHD Self-Report Scale (ASRS) was given to the Harvard recruits. This screener is a powerful tool used to discriminate DSM-IV cases from non-cases for screening purposes [49] and has been found to outperform much lengthier questionnaires for this purpose [50]. ASRS has been shown to be significantly related to the comparable clinical symptom ratings for inattention and impulsivity-hyperactivity, but to vary substantially in concordance (Cohen's k in the range 0.16–0.81). Harvard volunteers who scored above 3 on the ASRS were excluded from the study. All participants had vision that was normal or corrected to normal.

**Table 1:** Behavioral data for Landmark College participants (SR group).

	EXP			WAIS (or WISC)			READING					CTOPP
ID Code	1	2	3	Full Scale IQ	Verbal IQ	Perfm IQ	Test Name	Spelling	Single word reading	Nonsense word reading	Reading Compre-hension	
A	X	X		85	91	78	WIAT II	64	54	58	64	
B	X	X		101	98	105	WIAT II	84	66	80	100	
C	X	X		86–90			WJIII	56	54	72		
D	X	X		94			WJIII		65		83	
E	X	X		97	94	100	WJIII	62	57	67	96	
F	X	X		97			KTEA		1	2	2	
G	X	X		117	100	125	WJIII	79	99	94		
H	X	X		**								
I	X			86	100	73	WJIII	Average	Average	Superior		
J		X			110	100	WJ-Munoz		86			
K	X		X	110	111	109	WIAT	102	108	94		PA:94 RAN:61
L			X	89	82	100	WRAT	80	75			
M			X	131	103	124	WRAT/*WJIII	99	86	91*	108*	
N			X	108	105	110	WIAT	63	76	84	100	PA: 85 PM:76 RAN:46
O			X	80	82	83	WIAT II	51	65	61	63	

Abbreviations:

WRAT Wide Range Achievement Test.

WIAT Wechsler Individual Achievement Test.

WJIII Woodcock Johnson Achievement Test III.

WJ-Munoz Woodcock Munoz Spanish Achievement Test.

KTEA Kaufman Test of Educational Achievement (percentiles).

WJR Woodcock Johnson Reading.

CTOPP Comprehensive Test of Phonological Processing.

PA Phonological Awareness.

PM Phonological Memory.

RAN Rapid Automatized Naming.

Perfm Performance IQ.

* Indicates Nonsense Word and Comprehension from WJIII (other scores from WRAT).

** French narrative report without numerical data.

#### Apparatus

Stimuli were presented on a 20-inch Apple Cinema flat-screen LCD monitor viewed at a distance of 70 cm, with a resolution of 1680 by 1050 pixels and a refresh rate of 60 Hz. Stimulus presentation and data acquisition were controlled by custom software using Matlab (The Mathworks, Natick, Massachusetts), and the Psychophysics Toolbox [51]. Participants used a chinrest with a forehead bar to stabilize the head.

#### Stimuli

**Figure 2:**
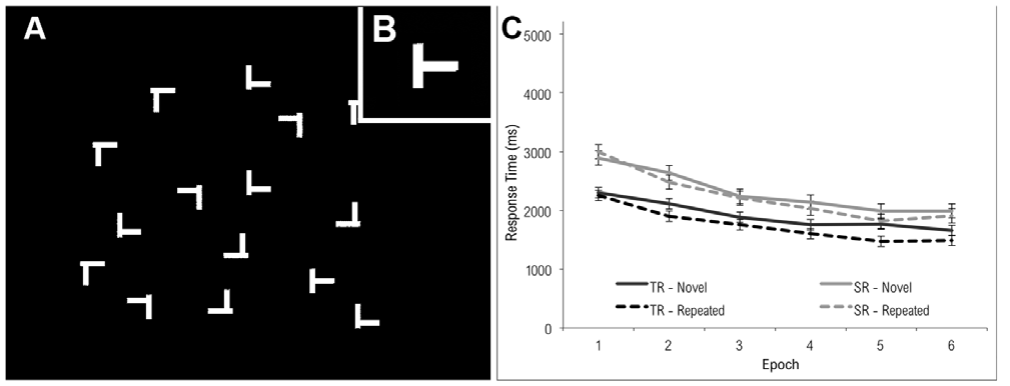
Example of stimulus used in Experiment 1. Participants search for and identify a side-facing T-shaped target, and indicate its direction left or right. The spatial context (A) is defined by a field of L-shaped objects, doglegged to resemble the target (B), inset. Manual response times for Experiment 1 are plotted in (C). Both typical readers and individuals with dyslexia show progressively faster search times in both the repeated and novel conditions, revealing a well-known practice effect associated with this task. Response times for repeated trials are generally shorter than for novel trials, and this difference grows as a function of epoch, indicative of a contextual cueing effect ascribed to spatial learning. The contextual cueing effect was equivalent for both SR and TR groups.

Search displays consisted of twelve white objects (luminance: 360 cd/m^2^) on a black background (3 cd/m^2^), see Figure 2. One of these objects, which served at the search target, was the letter ‘T’, tilted by 90° either to the left or to the right. The direction of the tilt was randomly chosen, with the constraint that each direction occurred in exactly half of the displays. The other eleven objects were shaped like the letter ‘L’ in one of four randomly chosen orientations (0°, 90°, 180°, or 270°). Following Chun and Phelps [52], one leg of the L was offset to increase its similarity with the target. The objects had diameters of approximately 1.6° of visual angle and were randomly distributed on a display area of 22° by 22°. The minimum distance between the centers of neighboring objects was 3.8°.

#### Procedure

**Figure 3:**
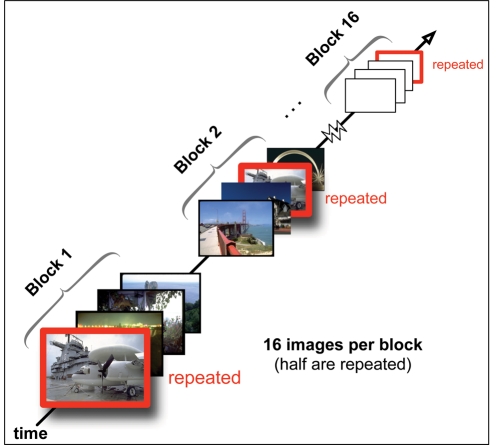
Contextual cueing paradigm. Participants search for a target in an image, and response times are observed. The paradigm assumes that spatial memory of the scene facilitates search for scenes that are repeated. A contextual cueing effect is observed if search is progressively speeded for repeated scenes compared to novel scenes as a function of block [7]. Here, the block design used in Experiments 2 and 3 is illustrated, consisting of 16 blocks, each containing 8 novel and 8 repeated images, randomly arrayed. (Blocking in Experiment 1 differed, employing 30 blocks of 24 trials, with 12 repeated images per block.).

Twelve stimulus displays were chosen to serve as ‘repeated displays’ (repeated) and were presented to the subjects multiple times, while all other displays were ‘new displays’ (novel) that were shown only once. Each subject saw the same sets of repeated and new displays, but in individually randomized order. Subjects performed 30 blocks of 24 search trials. Each block consisted of a random sequence of the same twelve repeated displays that were shown in every block, plus twelve new displays. The subjects' task was to find the target and press the ‘Z’ button or the ‘M’ button on a computer keyboard if the target was tilted to the left or to the right, respectively. They were instructed to perform this task as quickly and as accurately as possible, but no time limit was imposed on their responses, and they were allowed to move their eyes freely throughout the experiment. After their manual response, subjects were presented with a sound informing them about whether their response was correct (800 Hz tone played for 20 ms) or incorrect (400 Hz tone played for 100 ms). Subsequently, a blank black screen was shown for 1 s, followed by the next trial. Subjects took short breaks after each block and started the next one with a mouse click. No information regarding block structure or scene repetition was given to participants. (The general principles underlying the experimental design are illustrated in Figure 3).

### Results

#### Analysis

In terms of responses made, those in TR incorrectly identified the target on 1.5% of trials while those in SR incorrectly responded on 1.8% of trials [*t* (df) = 1.06, p = 0.30]. These trials were excluded from the analyses. Owing to the relatively low complexity of the task, no time cut-off was used, and none of the trials had a “no response.”

The dependent variable was Response Time (RT) in milliseconds. The 30 trial blocks were collapsed into 6 equal-sized epochs for analysis. A repeated-measures mixed model analysis of variance (ANOVA) was conducted to compare RTs across group (SR, TR), trial type (novel, repeated), and epoch (1–6). There were significant main effects of group, *F*(1, 27) = 10.86, *p*<0.01, trial type, *F*(1, 27) = 39.51, *p*<0.0001, and epoch, *F* (5, 135) = 208, *p*<0.0001. SR subjects had slower RTs (*M* = 2278 ms) than did TR subjects (*M* = 1830 ms); the repeated items were detected faster (*M* = 1995 ms) than the novel items (*M* = 2114 ms); and RTs generally decreased over the course of the experiment (epoch 1: *M* = 2612 ms; epoch 6: *M* = 1762 ms).

A significant interaction between trial type and epoch, *F*(5, 135) = 3.91, p<0.01, indicated that the difference in response time between novel and repeated items became larger during the second and later epochs. This was to be expected—and is typical for a contextual cueing effect–because, at the outset, every item was novel, and the effect of repetition would grow more salient over the course of the experiment. A second significant interaction was found between group and trial type, *F*(1, 27) = 5.96, p<0.05. The response time difference between the novel and repeated items was more pronounced for the TR group than for the SR group. That is, the benefit of repetition was greater for the TR than for the SR group. Thirdly, group and epoch were found to interact significantly, *F*(5, 135) = 8.88, *p*<0.0001, indicating that the gap in response time between SR and TR subjects became narrower during the course of the experiment (i.e., the drop in response time was steeper for the SR than for the TR group, indicating a greater overall learning effect for the SR group). The three-way interaction, however, was not significant. This finding is consistent with previous observations in dyslexia [14].

The observation that the SR group is slower overall at visual search is consistent with prior studies suggesting focal visual attention deficits in dyslexia [26]. Impairments in visual search can lead to deficits in contextual cueing [53]. Therefore, it is perhaps surprising that we do not observe deficits for spatial learning in the SR group. Despite deficits for search, search benefits due to contextual cueing were observed in both groups, and the extent of this spatial learning benefit was indistinguishable in these groups, opening the possibility that scene perception and/or scene memory is enhanced in the SR group.

## Experiment 2: Real-World Scenes

This experiment investigated the hypothesis that contextual cueing effects are enhanced in the SR group when spatial contexts are natural scenes. In Experiment 1, contextual cueing was investigated for contexts composed of letter-like objects. There, spatial learning was biased to information local to the target [9]. The situation is different when contextual cueing is performed in natural scenes. Here, long-range interactions are more important, and information global to the scene is integrated in spatial learning [10]. Long-range visuospatial processing is reported to be enhanced in dyslexia [27], [29], [36]. Therefore, we expect that contextual cueing is also likely to be enhanced in dyslexia for contexts that are natural scenes.

### Methods

#### Participants

Two groups of subjects participated in Experiment 2. One group consisted of 9 students with histories of reading struggles randomly drawn from the pool of volunteers from Landmark College described in Experiment 1; the other consisted of 8 students with typical reading abilities at Harvard University as before. Other criteria were as in Experiment 1.

#### Stimuli and Apparatus

**Figure 4:**
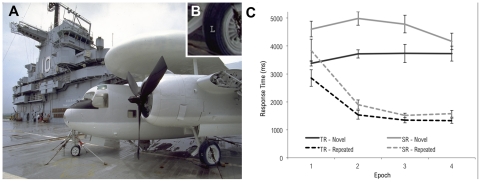
Example of stimulus used in Experiment 2. (A) Participants search for and identify a T or L target letter (here, a letter “L” on the aircraft tire, lower right). (B) Inset detail reveals target location. (C) Results of Experiment 2. Both typical readers and individuals with dyslexia show progressively faster search times for repeated trials, but not for novel trials. The magnitude of this difference was equivalent for both groups.

Stimuli consisted of photographs of real-world scenes previously used by Brockmole and Henderson [8]. Each scene (subtending a visual angle of 32° by 23°) contained a single gray T or L 0.18° in height (Figure 4b). Stimuli were displayed at a resolution of 800×600 pixels on a 19-inch CRT display at a constrained viewing distance of 70 cm. A video game controller was used to collect responses. Participants used a chinrest with a forehead bar to stabilize the head.

#### Procedure

Two types of trials were created. To measure baseline search speed, novel trials presented an image that had not been previously shown in the experiment. Any decrease in search speed on novel trials over the course of the experiment is therefore attributed to general practice effects. In contrast, repeated trials presented one of eight images that were previously displayed. With each repetition of an image, target location, but not target identity, was held constant. If this contingency is learned, search can be more efficient and response times on repeated trials should therefore decrease at a faster rate than observed on novel trials (i.e., an effect of learning over and above practice). The trial sequence was composed of 16 blocks, each containing 8 randomly selected novel trials and the 8 repeated trials (see Figure 3).

Trials were self-paced. Participants began each trial by fixating a dot in the center of the screen and pressed a button to initiate the onset of the stimulus. Targets were identified by pressing one of two buttons corresponding to the target (L or T). Trials were terminated if a response was not made within 20 s. No information regarding block structure or scene repetition was given to participants.

### Results

#### Analysis

TR participants failed to respond on 3.8% of trials while those in the SR group did not respond on 9.6% of trials [*t*(15) = 6.05, *p*<.001]. However, this difference between groups was driven by the novel trials where the TR group failed to respond on 7.3% of trials and the SR group failed to respond on 16.4% of trials. This is a byproduct of overall slower search among SR participants (more below). By including an upper bound on response times in the study's design, the baseline rate of search for the SR group is likely to be underestimated in our data. Concerning the repeated trials (those subject to learning), however, both groups responded on more than 97% of trials. Thus, the impact of trial repetition on RTs is accurately described. The statistical consequence of these data patterns, if anything, is an underestimation of contextual cueing for the SR group. We are therefore at a disadvantage to support our hypothesis that SR participants will show bigger contextual cueing effects than TR participants. Accuracy was uniformly high with incorrect responses occurring on 1.2% of trials for the TR group and 2.1% of trials for the SR group [*t*(15) = 1.96, *p* = .07]. Trials on which a response was incorrect were excluded from the analyses.

The 16 trial blocks were collapsed into 4 equal-sized epochs for analysis. Search times were submitted to a 2 (group: TR vs. SR) ×2 (trial type: repeated vs. novel) ×4 (block) mixed model ANOVA (see Figure 4c). All three main effects were reliable [group: *F*(1, 15) = 13.1, *p*<.01; trial type: *F*(1, 15) = 397, *p*<.001; epoch: *F*(3,45) = 14.7, *p*<.001]. As in Experiment 1, SR observers took longer (*M* = 3421 ms) to find the target than those in TR (*M* = 2701 ms); faster responses were elicited on repeated trials (*M* = 1997 ms) compared with novel trials (*M* = 4165 ms), and RTs decreased over epochs (*M*'s = 3701 ms, 3057 ms, 2862 ms, and 2707 ms across epochs 1–4, respectively). The interaction between trial type and epoch was also observed [*F*(3, 45) = 31.2, *p*<.001]. Whereas search times for novel trials did not change over blocks (linear trend: *F*(1, 16)<1), responses to repeated trials progressively decreased over the course of the experiment (linear trend: *F*(1, 16) = 66.1, *p*<.001). Critically, the three-way interaction was not reliable [*F*(3, 45) = 1.14, *p* = .34], indicating equivalent contextual cueing among SR and TR groups. The hypothesis that contextual cueing is enhanced in the SR group for contexts composed of natural scenes was not supported, as the search benefit due to contextual cueing was indistinguishable between the two subject groups.

Search deficits (in terms of overall longer response times) are clearly evident in the SR group. This raises the question of whether search latencies observed in dyslexia result from deficits in visual attention [26], or visuomotor deficits that slow response [54]. Inspection of Figure 4 suggests that latencies in visual search (defined as the magnitude of the difference in RT between the SR and TR groups) that are clearly evident at the beginning of the task are substantially reduced toward the end of the task, after practice and contextual cueing have had an effect. If contextual cueing reduces search latencies over the course of the experiment, this provides evidence that the slower response in dyslexia is likely the result of deficits in visual attention, and is not expressly due to a lag in visuomotor reaction as proposed, because such visuomotor deficits would not benefit from the memory-guided search.

To shed more light on this question, even though the corresponding overall interactions did not reach significance level, we employed a more focused approach and analyzed the RTs only at the end of the experiment (epochs 3 and 4), when contextual cueing, motor rehearsal, and practice with the task were expected to have had maximal effect. For novel trials in epochs 3 and 4, the main effect of group was significant, *F*(1, 15) = 4.41, *p* = .05. The repeated trials in the same blocks failed to show a similar effect of group, *F*(1, 15) = 2.58, *p* = .13 (see Table 2). This indicates that the SR group was still slower than the TR group at the end of the experiment when it came to searching through novel trials, but that, when it came to repeated trials, the differences between groups were so small at the end of the experiment that they failed to reach significance. This suggests some support for the hypothesis that, in the natural scene context, search latencies among struggling readers are largely eliminated by contextual cueing. It may, therefore, be that visuospatial deficits, and not motor response deficits, are the predominant factor accounting for search delays among struggling readers, when searching real-world scenes.

**Table 2:** Search latencies after contextual cueing and practice.

	Novel		Repeated	
Experiment	Latency	% of TR	Latency	% of TR
1	312	18	394	26
2	789	22	214	16
3	1223	72	583	51

**Abbreviations:** Latency = mean RTs for SR group minus mean RTs for TR group. % of TR = latency as a proportion of mean RTs for TR group, expressed as percentage. All means computed for the latter half of the experiment only (epochs 4–6 for Exp 1; blocks 9–16 for Exps 1 and 2).

## Experiment 3: Low-Pass Filtered Scenes

Here we explore contextual cueing in natural scenes that are low-pass filtered. Evidence suggests that dyslexia is linked to advantages, compared with the performance of typical readers, for visual cognition invoking long-range processing involving use of information the periphery [27], [29], [55]. Therefore, in this experiment, the application of a low-pass filter to natural scenes serves to limit the efficacy of foveal vision, and thereby biases visual processing toward peripheral visual content for which strengths are reported in dyslexia. This leads to the prediction that contextual cueing will be stronger in the SR group.

### Methods

#### Participants

A group (SR) of 5 students with dyslexia was recruited from Landmark College, and a control group (TR) included 10 students from Harvard University, as previously described. None of the volunteers in this experiment participated in Experiment 2.

#### Stimulus, Apparatus, and Procedure

**Figure 5:**
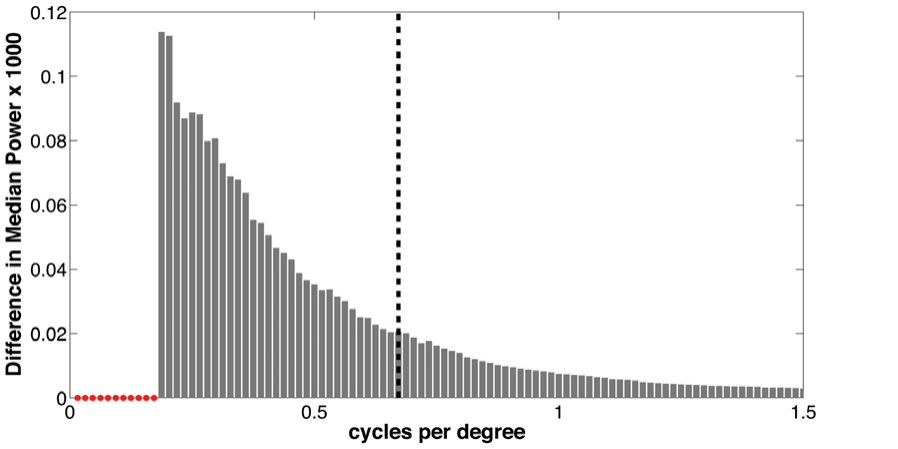
The effects of low-pass filtering on spectral power. Power spectra in 800 frequency bins were computed for the unfiltered images used in Experiment 2, and compared to power spectra of the Gaussian filtered images used in Experiment 3. The median difference in power per bin is plotted. This shows that seventy-five percent of the difference is accounted for in frequency bins between 0.2 and 0.67 cycles per degree (left of vertical dashed line). The power does not differ significantly in the range 0 to 0.19 cycles per degree (small red circles).

**Figure 6:**
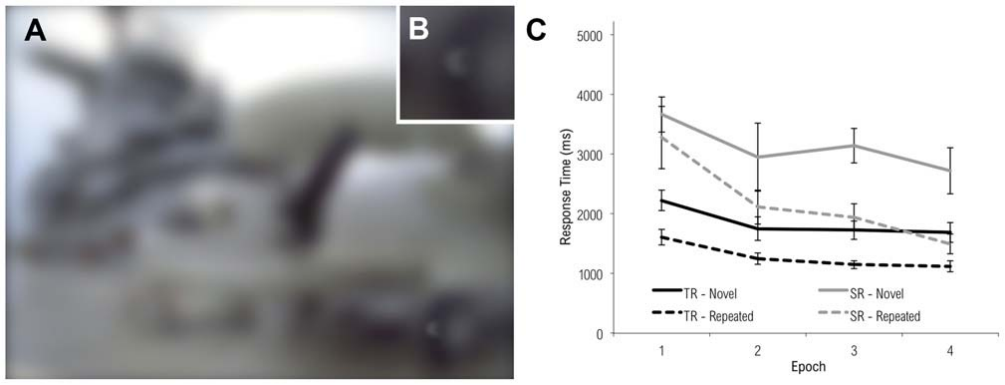
Example stimulus used in Experiment 3. (A) Participants search for a C or backwards C target (aircraft tire, lower right). Search times in novel and repeated contexts are compared to observe a contextual learning effect. (B) Inset detail reveals target location. (C) Results of reaction time analysis for Experiment 3. Individuals with dyslexia, but not typical readers, show evidence of contextual learning.

Stimuli consisted of low-pass filtered color photographs of real-world scenes. Here, Adobe Photoshop CS3 was used to apply a Gaussian filter of radius 13.5 pixels (.54 degrees) to the image set used in Experiment 2. To understand the effect of the filter, a power spectrum was computed for each of the 162 images used, before and after the filter was applied. A two-sample Kolmogorov-Smirnov test (alpha = 5%) was used to compare the difference in median power before and after filtering in each of 800 spectral frequency bins. No significant difference was observed for spatial frequencies lower than 0.19 cycles/degree. However, as expected, the filtering produced significant effects in spatial frequency components higher than this, with 75% of the difference in median power accounted for in spatial frequencies lying between 0.20 and 0.67 cycles/degree (see Figure 5). Each scene contained a normal or mirror reversed C whose visual diameter was 1.67° (Figure 6b) that was blurred using a Gaussian filter with a radius of 4.4 pixels (.18 degrees). Thus, a subtle contrast difference was apparent between targets and backgrounds. All other aspects of the apparatus and design were the same as in Experiment 2.

### Results

#### Analysis

TR participants failed to respond on <1% of trials while those in the SR group did not respond on 5.2% of trials [*t*(13) = 2.22, *p*<.05]. Once again, this difference between groups was driven by the novel trials where the TR group failed to respond on 1.4% of trials and the SR group failed to respond on 8.0% of trials. Concerning the repeated trials, both groups responded on more than 97% of trials. As in Experiment 2, therefore, we are at a disadvantage to support our hypothesis that SR participants will show bigger contextual cueing effects than TR participants. Accuracy was uniformly high with incorrect responses occurring on 2.2% of trials for the TR group and 2.6% of trials for the SR group [*t*(13)<1]. As before, trials on which a response was incorrect were excluded from the analyses.

As in Experiment 2, the 16 trial blocks were collapsed into 4 equal-sized epochs for analysis. Search times were submitted to a 2 (group: TR vs. SR) ×2 (trial type: repeated vs. novel) ×4 (epoch) mixed model ANOVA (see Figure 6c). As in Experiment 2, all three main effects were reliable [group: *F*(1, 13) = 19.6, *p*<.001; trial type: *F*(1, 13) = 57.9, *p*<.001; epoch: *F*(3, 39) = 21.7, *p*<.001]. Consistent with prior work, SR observers took longer to find the target (*M* = 2660 ms) than controls (*M* = 1563 ms); faster responses were elicited on repeated trials (*M* = 1588 ms) compared with novel trials (*M* = 2270 ms), and RTs decreased over epochs (*M*'s = 2432 ms, 1842 ms, 1803 ms, and 1637 ms across epochs 1–4, respectively). The statistical hallmark of contextual cueing, an interaction between trial type and epoch, was marginally reliable [*F*(3, 39) = 2.72, *p* = 0.58]. However, a reliable three-way interaction [*F*(3, 39) = 3.15, *p*<.05] was observed indicating that contextual cueing differed by group. This prompted us to consider the TR and SR groups separately. To more precisely characterize the individual groups, we examined performance across all 16 experimental blocks and therefore conducted separate 2 (trial type) ×16 (block) repeated measures ANOVAs. (Observed mean search times (ms) by group, block, and trial type are shown in Table 3.) In these analyses, both groups displayed faster response times to the repeated trials and on the later blocks (all p's<.01). However, the critical interaction between trial type and block was not observed in TR [*F*(15, 135) = 1.51, *p* = .11] while it was observed for SR participants [*F*(15, 60) = 1.92, *p*<.05]. That said, because the differential effect of block on novel and repeated trials was marginal among typical readers, we also used linear regression analysis as a way to estimate the rate of learning in each group. For typical readers, linear models indicated a 42 ms decrease in RT per block within the repeated trial condition. Comparatively, for struggling readers, these models indicated a 144 ms decrease in RT per block, a rate of learning triple that of typical readers. From this analysis too, then, the hypothesis that contextual cueing is enhanced in struggling readers when scene contexts are low-pass filtered is supported.

**Table 3:** Experiment 3 results: Mean RTs (ms) by group, block, and trial type.

	Block															
TrialType	1	2	3	4	5	6	7	8	9	10	11	12	13	14	15	16
	**Typical Readers (TR)**															
**Novel**	2564	1982	2435	1909	1617	1804	1725	1847	1581	1995	1383	1940	1462	1438	1797	2054
**Repeated**	2027	1697	1375	1324	1195	1385	1211	1203	1264	1119	1161	1045	1124	1140	1078	1130
	**Struggling Readers (SR)**															
**Novel**	3531	3286	3694	4132	3215	2717	1954	2912	2920	3788	2767	3086	2302	2541	2524	3503
**Repeated**	4127	3747	3296	1932	2388	2193	1883	1978	2188	2005	1757	1799	1333	1654	1591	1396

## Discussion

### Implications for Dyslexia

Figure 1 schematically summarizes the results. While no group differences in contextual cueing were observed for letter-like contexts (Experiment 1) or natural scenes (Experiment 2), college students who face lifelong struggles with reading exhibited advantages in building scene memory when scenes are defined by natural images that are low-pass filtered (Experiment 3). This supports the peripheral-bias hypothesis [37]: When a low-pass filter is used in natural scenes to limit the efficacy of foveal vision and thereby biases visual processing toward long-range content for which strengths are reported in dyslexia [27], [29], [55], spatial learning is enhanced in struggling readers.

To what extent do these results in struggling readers apply to people with dyslexia in general? Those in the SR group, who are enrolled in special a remedial college for people with language-based learning disabilities, have had lifelong histories of struggles with reading that are typically characteristic of dyslexia. However, dyslexia is not a unitary phenomenon, but is instead described by subtypes that are as yet not well understood [56], [57]. Given the generally small sample sizes used in our experiments, caution is therefore needed in generalizing our findings to all subtypes of dyslexia. Clearly, additional studies with large numbers of individuals, tracking factors indicative of subtypes within dyslexia, are needed. With this in mind, several explanations for our findings should be considered.

Given that the SR group consists of individuals who face unusual challenges with reading, it may not be dyslexia per se, but rather competition with reading that is the operant mechanism responsible for the effects observed. For example, tradeoffs between reading and visual cognition are reported in a functional magnetic resonance imaging study of illiterate adults who learn to read [58]. As acquisition of literacy strengthened activation in the left fusiform regions, activation for checkerboards and faces in the same location decreased. Similar effects of competition between reading and visual cognition are also observed in remediation studies of people with dyslexia [29], [31], [59]. These reported that as struggling readers developed strategies that improved their reading, previously observed enhancements in peripheral sensitivity were observed to diminish. Therefore, similarly, it is possible that the effects we observe result from neural plasticity linked to lifelong experience with reading, differentiated in the SR and TR groups. If this were the case, then adults with dyslexia who struggled with reading throughout their lives would be expected to show enhanced peripheral sensitivity, when compared with people with dyslexia who had the benefit of a successful reading intervention early in life, and this effect would be independent of subtype classification.

If, on the other hand, we accept that the SR group is linked to dyslexia, then a possible alternate explanation for the findings in Experiment 3 is that contextual cueing is enhanced simply because those in the SR group take longer to find the target, affording this group more time to commit the scenes to memory during search. Dyslexia is linked to impaired sensitivity to low spatial frequencies [60], for briefly flashed sinusoidal gratings at ∼4 cycles per degree. Diminished threshold sensitivity to low spatial frequency contrasts would make it difficult for these individuals to find the low spatial frequency targets, slowing their search and increasing their exposure to each scene. In Table 2 we summarize the mean search latencies in the last half of each Experiment, when contextual cueing and practice effects are the strongest. While in Experiments 1 and 2, the SR group spends on average about 20% more time searching each scene, in Experiment 3 the SR group spends 51% more time searching scenes that are repeated, and 72% more time searching those that are novel. The most pronounced latencies (observed in Experiment 3) are thus linked with enhanced contextual cueing (in that same experiment). Therefore it is plausible that the added time spent in search serves to facilitate scene learning, and future experiments should attempt to control for this effect.

### Rapid semantic characterization of ambiguous scenes

It has been shown that, in real-world scenes, shifts of attention are initially based on scene identity, and subsequent shifts are guided by more detailed information regarding scene and object layout [8]. Semantic memory (e.g., “the L is on the tire”) has been shown to play a causal and independent role in learning associations between objects in real-world scenes [61]. It has been demonstrated that many people with dyslexia are slower at retrieving names of letters, objects, and colors [62], deficits that are linked to a more generalized impediment in retrieving semantic labels from visual stimuli [63]. Difficulties retrieving verbal labels for objects and images are accompanied by a greater incidence of tip-of-the-tongue (TOT) responses, wherein the identity of the visual stimulus is familiar, understood, and known, but cannot be accurately named [64]. Anecdotally, people with dyslexia are observed to compensate for difficulties with semantic retrieval by making semantic substitutions in speech. Thus, if an individual is struggling to retrieve the verbal label for “tire,” the idea “the L is on the tire” might be conceptualized instead as “the L is on the black blob,” using a semantic substitution that is personal and meaningful to the individual, and that can be retrieved at speeds close to those seen in typical verbal retrieval.

We suggest that the compensatory practice individuals with dyslexia have with semantic substitution could account for the findings in Experiments 2 and 3. When natural scenes are low-pass filtered, object identity can become ambiguous. In this case, people in the SR group, who are adept at creating semantic labels for things that are difficult to describe will continue to use semantic substitution to generate meaningful semantic cues able to guide attention in contextual cueing. However, those in the TR group, who are likely expecting to apply accurate semantic labels for the low-pass filtered forms, would have difficulty doing so. Lacking semantic categories and object names to guide attention, the TR group would be at a disadvantage for contextual cueing when the scenes are low-pass filtered. But the two groups would be equivalent for the unfiltered contexts because semantic substitution used by the SR group and accurate verbal identification used by TR group would be equally effective for defining semantic cues able to guide attention. This hypothesis could therefore explain our findings indicating no significant group differences in contextual cueing for natural scenes (Experiment 2), but advantages for the SR group when scenes are low-pass filtered (Experiment 3). Empirically testing this semantic hypothesis should be the focus of future work.

### Motor impairments cannot fully explain response latencies observed

All three experiments revealed a significant main effect of group indicating that the SR group was overall slower at search, compared with the typical readers. Search requires focal attention [22], and search impairments in dyslexia have been cited as evidence of a deficit in the magnocellular pathways [26], well explained by associated focal attention deficits [16]–[19], [65], [66]. Thus, to the extent that the SR group is representative of dyslexia, our findings are consistent with the observed deficits in focal attention. However, an alternate explanation is that the slower reaction times are a consequence of impaired motor response in dyslexia linked to cerebellar abnormalities [54], and not an effect of focal visual attention deficits.

To investigate these possibilities we separately analyzed search latencies in the latter half of Experiment 2, after contextual cueing, motor rehearsal, and practice with the task have had a chance to express their fullest benefit. We found that in repeated trials, where search is guided in part by memory, latencies were small (214 ms) and could not be reliably measured in our experiment. But in the novel condition the latencies were substantial (789 ms) and significant, persisting despite benefits of practice and motor rehearsal accrued in the first half of the experiment. We expected that motor deficits would have the same effect for both the novel and repeated conditions. However, the effects of focal attention deficits were expected to be different in the novel and repeated cases, because focal attention plays a small role when trials are repeated, and search is guided by memory.

The analysis of mean RT in the latter half of Experiment 2 (see Table 2, and Results described previously) showed that search times for struggling readers were indistinguishable in our experiments from the typical readers when the scenes were repeated, suggesting that once the task had been practiced, and motor actions had been learned, motor deficits no longer played a significant role in impeding search. However, in the novel trials, significant latencies remained evident even after motor rehearsal and task learning. Therefore, our observations lend support to the hypothesis that visual attention deficits, and not motor deficits, are the dominant mechanism acting to impede search in struggling readers in real scenes.

### Spatial learning and distributed attention unaffected by attention deficits

In all cases, our experiments showed that, when search is performed in scenes that are novel, visual search was impaired in those who struggle to read. These observations support previous reports associating dyslexia with deficits for search [26], [67], a finding that is in turn consistent with observations associating dyslexia with deficits for focal attention [16]–[19], [65], [66]. In contrast, none of our experiments revealed corresponding deficits for contextual cueing. The fact that contextual cueing was unaffected by deficits for focal attention in dyslexia is consistent with research that shows that the rapid categorization of natural scenes proceeds in parallel with attention, and is not affected by focal attention loads [68]. Contextual cueing has also been shown to be robust against interference from working memory loads [69], [70], and thus working memory deficits in dyslexia [71] were not expected to interfere with processes important in scene learning. Consequently, visuospatial deficits characteristic of dyslexia were not expected to impair contextual cueing, and this was supported by our observations.

A number of authors stress a distinction between systems for focal attention and those for rapid distributed spatial attention, with both types of systems acting in concert to build the visual percept in a complex scene [21], [22]. Distributed attention is thought to play a role in contextual cueing to form a rapid initial hypothesis about the global scene layout that is later refined through search [72]. Numerous studies point to findings that focal attention and slow sustained attention are impaired in dyslexia [25], [26]. However, emerging research, including the findings here, suggests that rapidly deployed distributed attention is unimpaired in dyslexia, and if anything may be enhanced. For example, as mentioned before, recognition speed for impossible figures, a task that depends on the holistic integration of long-range spatial information across a scene, is observed to be enhanced in dyslexia without compromising speed [27]. Those with dyslexia have been observed to respond more rapidly to an unattended peripheral flash when the flash occurs at eccentricities >8° [35], [36]. Other studies found visuospatial advantages in dyslexia in letter identification tasks in cases where letters are flashed simultaneously at fixation and in the periphery, typically at eccentricities >8° [28]–[34], a task that requires rapid deployment of spatially distributed attention. Collectively, these studies link dyslexia to advantages for distributed forms of spatial attention, typically in circumstances where peripheral (eccentricities >∼8°) information is important.

### Implications for the magnocellular theory of dyslexia

The visual channels responsible for the processing of low spatial frequency information are thought to play a distinct role in the higher-order cognitive processing of visual information. Theories of visual recognition propose that visual scenes are processed in a temporal order that proceeds from coarse-to-fine [73]. Here, low spatial frequency information is thought to reach higher-order areas most rapidly to allow for an initial coarse parsing of the scene that precedes an analysis of high spatial frequency details [74]–[76]. An empirical test of this hypothesis showed that, when two spatial frequency-filtered natural scenes were presented in rapid succession during fMRI and ERP recording, low spatial frequencies increased activity in prefrontal and temporo-parietal areas first, before high spatial frequency detail produced an effect [77]. This demonstrates that low-pass signals can rapidly activate high-order areas to provide spatial, semantic, and attentional signals that together may promote perceptual organization and categorization of visual scenes.

Our findings that visual search is consistently impaired in the SR group are in line with reports of attention deficits and therefore support prior studies linking dyslexia to impairments in the magnocellular pathways [26]. However, if the findings of Experiment 3 are not simply consequences of longer exposure or rapid semantic characterization, as suggested earlier, but instead are a result of enhanced processing in dyslexia in the visual channels responsible for the coarse preprocessing of scenes, then our finding of enhanced spatial learning in low-pass filtered contexts presents a challenge for magnocellular theories of dyslexia. Low spatial frequencies are believed to be conveyed primarily by the magnocellular visual pathways [78], [79] and dyslexia has been associated, albeit controversially, with deficits in functions conveyed by these channels [80], [81]. Therefore, a magnocellular deficit cannot concurrently account for deficits in search and advantages for spatial processing unless each of these functions are sub-served by magnocellular neurologies that are distinct.

We suggest that a possible alternate perspective, consistent with the peripheral-bias hypothesis [37], is that, rather than an overall magnocellular deficit, there is a magnocellular *shift* in dyslexia toward the periphery. This is proposed to skew the distribution of magnocellular neurons (relative to parvocellular) retinotopically toward the periphery. This would have the effect of underrepresenting the magnocellular density near the fovea, but of over-representing the density toward the periphery. Such a pattern would thus concurrently account for magnocellular deficits near the fovea, including deficits for central-field tasks such as visual search [38], and for enhancements in spatial learning, conveyed by long-range interactions involving the periphery [10], [12].

The proposal that magnocellular phenomena in dyslexia are better described in term of a retinotopic shift directed toward the periphery may be supported by emerging theories in the field of genetics [82], [83]. These theories suggest that cytoarchitectonic abnormalities in dyslexia [80], [84]–[87] are induced by heritable errors linked to 14 candidate genes thought to regulate neuronal migration (see review [88]). These errors are thought to disrupt the development of visual and auditory regions in the thalamus and elsewhere, including the magnocellular lamina in the LGN. Given that the fovea is magnified and highly over-represented in the LGN, even a minor redistribution of magnocellular neurons directed toward the retinotopic periphery, as proposed here, would have observable eccentricity-dependent consequences in dyslexia. Future tests of magnocellular phenomena should explicitly segregate visual contributions in the inner fields (inside 8°) from those at larger eccentricities.

### Implications for education

The finding that college students with dyslexia are able to learn in some circumstances where typical readers cannot carries a number of important implications for education. It suggests that college students with dyslexia may benefit from encouragement in careers in which sensitivity to low spatial frequency scenes is valued. Radiology, astronomy, and cellular microscopy are all examples of domains making intensive use of spatial information in images that are blurred. Skills in processing blurred images may be beneficial also in other science-related fields. Furthermore, our findings show that contextual cueing is effective in counteracting latencies due to deficits in search, and therefore scene learning can serve as an effective compensatory technique for students with dyslexia. Practiced abilities for spatial learning can be used to spatially anchor memories for episodic information (such as names and dates). Such techniques have already been observed to effectively compensate for lapses in non-spatial memory in the elderly [89]. Similar gains can be expected in dyslexia.

Research in dyslexia has necessarily focused its greatest effort on phenomena pertinent to reading. Yet dyslexia has remained a puzzle for over a century. We suggest that efforts to investigate phenomena less relevant for reading, such as peripheral visual effects examined here, may be well rewarded. For not only do we believe that such research may help resolve questions that currently confound the interpretation of visual phenomena in dyslexia, but such research may lead to advancements in education that will increase support for children who otherwise struggle in school.
